# Testing the Effects of App-Based Motivational Messages on Physical Activity and Resting Heart Rate Through Smartphone App Compliance in Patients With Vulnerable Coronary Artery Plaques: Protocol for a Microrandomized Trial

**DOI:** 10.2196/46082

**Published:** 2023-10-02

**Authors:** Sayan Mitra, Cynthia M Kroeger, Jing Xu, Leah Avery, Andrius Masedunskas, Sophie Cassidy, Tian Wang, Imre Hunyor, Ian Wilcox, Robin Huang, Bibhas Chakraborty, Luigi Fontana

**Affiliations:** 1 Central Clinical School Faculty of Medicine and Health The University of Sydney Camperdown Australia; 2 Charles Perkins Centre The University of Sydney Camperdown Australia; 3 Office of Education Duke-National University of Singapore Medical School Singapore Singapore; 4 Program in Health Services and Systems Research Duke-National University of Singapore Medical School Singapore Singapore; 5 Centre for Quantitative Medicine Duke-National University of Singapore Medical School Singapore Singapore; 6 School of Health & Life Sciences Teesside University Tees Valley, England United Kingdom; 7 Department of Cardiology Royal Prince Alfred Hospital Sydney Australia; 8 Central Sydney Cardiology Royal Prince Alfred Medical Centre Sydney Australia; 9 School of Computer Science The University of Sydney Darlington Australia; 10 Department of Statistics and Data Science National University of Singapore Singapore Singapore; 11 Department of Biostatistics and Bioinformatics Duke University Durham, NC United States; 12 Department of Endocrinology Royal Prince Alfred Hospital Sydney Australia; 13 Department of Clinical and Experimental Sciences Brescia University Brescia Italy

**Keywords:** adherence, coronary artery disease, coronary heart disease, digital health, health behavior, heart rate, messages, mhealth, microrandomized trial, mobile app, physical activity, smartphone, telehealth, user motivation

## Abstract

**Background:**

Achieving the weekly physical activity recommendations of at least 150-300 minutes of moderate-intensity or 75-150 minutes of vigorous-intensity aerobic exercise is important for reducing cardiometabolic risk, but evidence shows that most people struggle to meet these goals, particularly in the mid to long term.

**Objective:**

The Messages Improving Resting Heart Health (MIRTH) study aims to determine if (1) sending daily motivational messages through a research app is effective in improving motivation and in promoting adherence to physical activity recommendations in men and women with coronary heart disease randomized to a 12-month intensive lifestyle intervention, and (2) the time of the day when the message is delivered impacts compliance with exercise training.

**Methods:**

We will conduct a single-center, microrandomized trial. Participants will be randomized daily to either receive or not receive motivational messages over two 90-day periods at the beginning (phase 1: months 4-6) and at the end (phase 2: months 10-12) of the Lifestyle Vulnerable Plaque Study. Wrist-worn devices (Fitbit Inspire 2) and Bluetooth pairing with smartphones will be used to passively collect data for proximal (ie, physical activity duration, steps walked, and heart rate within 180 minutes of receiving messages) and distal (ie, change values for resting heart rate and total steps walked within and across both phases 1 and 2 of the trial) outcomes. Participants will be recruited from a large academic cardiology office practice (Central Sydney Cardiology) and the Royal Prince Alfred Hospital Departments of Cardiology and Radiology. All clinical investigations will be undertaken at the Charles Perkins Centre Royal Prince Alfred clinic. Individuals aged 18-80 years (n=58) with stable coronary heart disease who have low attenuation plaques based on a coronary computed tomography angiography within the past 3 months and have been randomized to an intensive lifestyle intervention program will be included in MIRTH.

**Results:**

The Lifestyle Vulnerable Plaque Study was funded in 2020 and started enrolling participants in February 2022. Recruitment for MIRTH commenced in November 2022. As of September 2023, 2 participants were enrolled in the MIRTH study and provided baseline data.

**Conclusions:**

This MIRTH microrandomized trial will represent the single most detailed and integrated analysis of the effects of a comprehensive lifestyle intervention delivered through a customized mobile health app on smart devices on time-based motivational messaging for patients with coronary heart disease. This study will also help inform future studies optimizing for just-in-time adaptive interventions.

**Trial Registration:**

Australian New Zealand Clinical Trials Registry ACTRN12622000731796; https://www.anzctr.org.au/Trial/Registration/TrialReview.aspx?id=382861

**International Registered Report Identifier (IRRID):**

DERR1-10.2196/46082

## Introduction

### Background and Rationale

Physical activity has been widely recognized as an effective intervention for improving cardiometabolic health [1,2]. Regular endurance exercise has been shown to reduce body weight and visceral adiposity, improve insulin sensitivity and glucose tolerance, lower blood pressure, and enhance lipid metabolism, all of which contribute to the prevention and management of atherosclerotic cardiovascular disease [3-6]. However, achieving sufficient compliance with physical activity recommendations poses a significant challenge, which can be attributed to various factors, including lack of motivation, skills, resources, and limited access to exercise facilities, among others [7].

The emergence of new technologies and digital products such as wearable devices, mobile apps, and mobile health (mHealth) platforms offers the potential for the delivery of customized educational and motivational material, which may increase compliance with physical activity interventions [8]. Smartphones and mobile apps are the first and second most frequently used digital products in clinical research [9], and with other sensors connected to digital platforms, are increasingly becoming an integral part of daily health care routine [10].

Passive monitoring of health parameters can be accomplished using wearable devices, which allow for nonintrusive data collection [11]. These technologies allow for the gathering of real-time data, the tracking of progress, and the delivery of messages that are timed to align with a person’s daily routines and patterns of activity. This can provide a personalized and targeted approach to physical activity, which can help individuals be more motivated and engaged in regular physical activity [12,13]. To explore this further, we have designed a microrandomized trial (MRT) to investigate the impact of delivering motivational messages at different times of the day targeting physical activity compliance, duration, and intensity.

An MRT is a cutting-edge trial design for mHealth research. A unique feature of an MRT is that an individual can be repeatedly randomized multiple times throughout the duration of the study [14,15]. The overall aim is to fine-tune the mHealth intervention by analyzing the effects of the different intervention components (eg, behavioral prompts) on the activities of the participant (eg, physical activity compliance; Textbox 1). This random sequence within and between participants constitutes an MRT [16,17], with most MRTs contributing to the development of just-in-time adaptive interventions (JITAIs). JITAIs aim to deliver appropriate support tailored to individuals’ needs at the precise moment it is needed [18,19].

**What are the benefits of randomizing a participant multiple times over the duration of this trial?**

Microrandomized trials (MRTs) are unique clinical trial designs suitable for studying interventions using mobile health technologies. They randomize interventions at multiple time points, allowing for dynamic treatment investigation. Key features include fine-grained granularity, personalization, and sequential decision-making. MRTs provide additional information such as temporal dynamics, contextual effects, individual response patterns, and treatment adaptation. These trials offer insights into personalized and effective interventions in the mobile health context.

The unique experimental design of an MRT allows for capturing data with the aim of developing sharp decision-making tools or rules for fine-tuning the sending of messages as notifications. The ability to randomize a participant multiple times in an MRT gives it its distinctive characteristic, which is not present in a traditional parallel-group randomized control trial, thereby allowing us to study:

- the effects of the intervention multiple times over a given time period;
- if the motivational messages sent as notifications have any near-term effect on engagement with the intervention components over the duration of the study;
- if this near-term effect changes over the two 90-day study periods; and
- if the effects of the motivational messages in phase 1 are similar to those in phase 2, thereby having a long-term effect.

Through analyzing the data from above, researchers will be able to optimize just-in-time adaptive interventions more effectively for notification delivery through apps encouraging physical activity in patients with coronary heart disease.

The main purpose of the Messages Improving Resting Heart Health (MIRTH) study is to determine if (1) sending daily customized motivational messages through a research app improves motivation and engagement with physical activity recommendations in men and women with coronary heart disease randomized to a 12-month intensive lifestyle intervention, and (2) the time of the day when the message is delivered impacts compliance with exercise training. This trial will be a crucial step in determining the potential of technology to support people in their efforts to maintain a physically active lifestyle, which can have a significant impact on the prevention and management of cardiometabolic abnormalities and atherosclerotic cardiovascular disease.

The primary objective of this study is to evaluate the immediate effectiveness of motivational messages aimed at promoting physical activity in adults with stable atherosclerotic cardiovascular disease. The secondary objectives include assessing the impact of message delivery time (ie, 6 AM, 11 AM, or 3 PM) on the immediate effectiveness of the intervention and investigating its long-term effectiveness over a 12-month period by comparing results at the midpoint and end of the study.

The primary hypothesis is (1) that participants will demonstrate an increase in steps taken within 180 minutes of receiving motivational messages compared to those who do not receive the messages. The secondary hypotheses are (2) that participants will exhibit an increase in physical activity duration and heart rate within 180 minutes of receiving motivational messages compared to those who do not receive the messages; (3) the proximal effect sizes of message are different at different times of a day (ie, 6 AM, 11 AM, or 3 PM); and (4) that there will be no difference in long-term outcomes between phase 1 (months 4-6 of the Lifestyle Vulnerable Plaque Study [LIVEPLUS]) and phase 2 (months 10-12 of the LIVEPLUS) of the study.

### Previous Work

In the HeartSteps MRT study, research volunteers randomly received, through an app, contextually tailored physical activity notifications delivered up to 5 times per day at user-selected times [20]. During the 6-week trial, the implementation of a physical activity notification resulted in a significant 24% increase in average step count compared to no suggestion. However, the effect was not consistently maintained throughout the study, with higher compliance observed primarily at the beginning. Similarly, the 6-week DIAMANTE student study [21] demonstrated that motivational text messages based on a cognitive-behavioral approach led to a significant increase in daily step count, although the effect diminished over time. These findings highlight the need for further research to investigate the potential advantages of incorporating personalization and contextualization in text-messaging interventions, aiming to enhance the effectiveness of physical activity promotion [21]. Aguilera et al [22] explored the effects of text messages in alleviating depression and anxiety symptoms during the COVID-19 lockdowns in the United States in the StayWell at Home study. The study revealed a positive association between engagement in a text-messaging program rooted in cognitive-behavioral principles and the amelioration of depression and anxiety symptoms associated with the COVID-19 pandemic. The program involved the delivery of 2 messages per day over a period of 60 days and demonstrated significant engagement and effectiveness in enhancing mental well-being. These findings suggest that text-messaging interventions, when used as a standalone component of a comprehensive program, have the potential to serve as a valuable tool in public health initiatives targeting mental health concerns, facilitating improved health outcomes through increased engagement with the intervention.

## Methods

### Study Design

#### Overview

**Figure 1:**
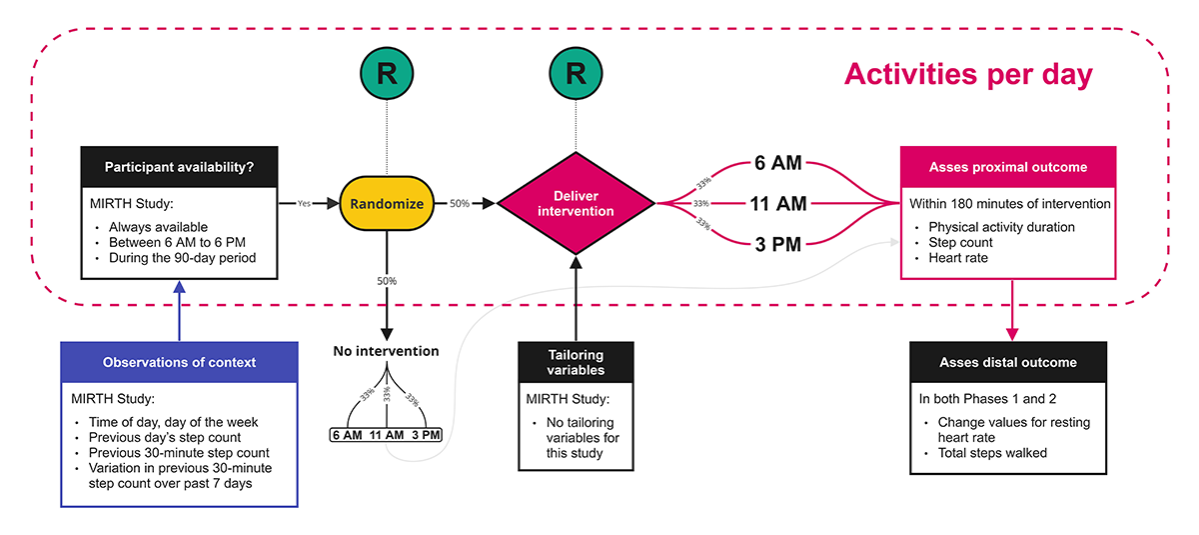
Microrandomized trial design for the Messages Improving Resting Heart Health (MIRTH) study. Intervention randomization is followed by time randomization, as shown by R. Figure adapted from Golbus et al [23].

**Figure 2:**
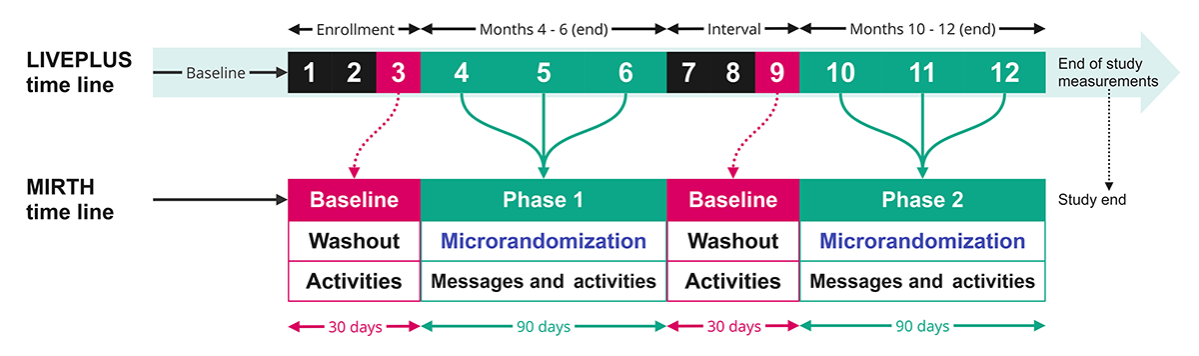
Time line of the Messages Improving Resting Heart Health (MIRTH) study, illustrating the participant flow and duration for each phase aligned with the Lifestyle Vulnerable Plaque Study (LIVEPLUS) time line. Baseline data collection for the MIRTH study will occur over a period of 1 month (30 days) at the ends of months 3 and 9 of the LIVEPLUS intervention. The collected data will include various metrics such as the average daily step count, duration, and heart rate from 6 AM to 6 PM. Additionally, we will also collect the average weekly physical activity duration. Activities refer to the LIVEPLUS component of the intervention (end-of-study measurements assessed at the end of month 12).

Figure 1 [23] provides an overview of the study design for the MIRTH MRT study. The intervention period consists of two 90-day periods, which are condensed into phases for the purposes of this protocol. Phase 1 corresponds to months 4-6 of the LIVEPLUS program, while phase 2 corresponds to months 10-12 (Figure 2). Throughout the study, participants are randomly selected at decision time points, which occur at 3 different times of the day: early morning (6 AM), late morning (11 AM), and early afternoon (3 PM). At the designated time point, each participant is randomly assigned to either receive or not receive the intervention component (motivational message) using a simple randomization method (Multimedia Appendix 1). The randomization process is independent of the participant’s allocation from the previous day. To ensure allocation concealment, a central randomization algorithm is used. Computerized sequence generation will be used to conduct participant randomization. Given the nature of the intervention, participants will be aware of the messages they receive. Subsequently, the duration and type of physical activity performed will be evaluated. A research personnel will assist participants in downloading the study app, creating a profile, and providing a brief demonstration of the app’s various functionalities. Detailed written instructions, along with a video tour of the app, can be accessed in the app’s help section. Screenshots of the app are available in Multimedia Appendix 2.

#### Study Population

The study will recruit adults aged 18-80 years with stable heart disease who have undergone a clinically indicated coronary computed tomography angiography scan at Royal Prince Alfred Hospital in Sydney, New South Wales, Australia, as previously described [24]. Only individuals with a confirmed positive diagnosis of a low attenuation plaque (quantifiable plaque within the –30 to 150 HU range) by 2 independent clinicians or researchers will be contacted for potential participation. Before collecting any participant data, informed written consent will be obtained. Volunteer self–opt-in will not be considered eligible for participation in the study.

#### Key Inclusion and Exclusion Criteria

The criteria listed in Textbox 2 were followed in selecting participants for the study.

**Inclusion and exclusion criteria.**

**Inclusion criteria:**

- Intervention group participants from the Lifestyle Vulnerable Plaque Study (Australian New Zealand Clinical Trials Registry ACTRN12620001151921)
- Adults who have access to and can competently use a smartphone (running iOS or Android)
- Presence of low-attenuation plaque on coronary computed tomography angiography
- aged 18-80 years
- BMI≥22.0 kg/m^2^
- Have no contraindications for the Lifestyle Vulnerable Plaque Study
- Able to provide full, informed consent

**Exclusion criteria:**

- History or clinical manifestation of any other significant metabolic, hematologic, pulmonary, cardiovascular, gastrointestinal, neurologic, immune, hepatic, renal, or urologic disorders, or cancer that, in the opinion of the investigator, would make the candidate ineligible for the study
- Non–magnetic resonance imaging–compatible implanted devices or implants
- Estimated glomerular filtration rate less than 30 mL/kg/1.73 m^2^
- Inability to exercise through a supine ergometer
- Claustrophobia
- Contraindications for adenosine: sinus node disease (eg, sick sinus syndrome and symptomatic sinus bradycardia), second- or third-degree heart blocks, unstable angina, bronchospasm (eg, asthma), heart transplant recipient, and history of seizure disorder
- Contraindications for glyceryl trinitrate: known nitrate hypersensitivity, severe anemia, severe aortic or mitral stenosis, and hypotension defined as resting systolic blood pressure equal to 89 mm Hg
- Not suitable for computerized tomography coronary angiography due to contraindications
- Psychiatric or behavioral problems (history of drug and alcohol abuse, eating disorder, etc)
- Breastfeeding or pregnant women, or those intending to become pregnant before the scheduled end of the intervention
- Concurrent participation in any other interventional study

#### Recruitment

The subsample for this study will be recruited from the intervention group of the LIVEPLUS (Australian New Zealand Clinical Trials Registry ACTRN12620001151921). Participants will be recruited from Central Sydney Cardiology and the Departments of Cardiology and Radiology at the Royal Prince Alfred Hospital in Camperdown, New South Wales, Australia. Eligible patients will be introduced to the study by their cardiologist. The research team will conduct a 30-minute informal web-based (Zoom) call to illustrate the study, which will include a presentation and an opportunity for participants to ask questions.

#### Baseline Assessment

The collection of participants’ physical activity data will occur in a free-living setting, reflecting their daily life activities. The baseline assessment for enrollment into the MIRTH study will be conducted based on the baseline assessment previously described for the LIVEPLUS [24].

#### Trial Arms

In contrast to traditional randomized controlled trials, the MRT approach in this study enables each participant to serve as their own control. As a result, there is a single group of research participants who are randomly assigned on a daily basis to either the intervention group (receive message) or the control group (no message) in order to provide data.

#### Intervention

Participants in the study will receive messages as app notifications based on a random schedule. Each day during the intervention period, participants will be randomized to either receive or not receive the messages at a designated time point. Some of the messages included in the intervention have been previously used in the DIAMANTE study [25]. Additional messages were created in collaboration with a health psychologist (LA) who specializes in health behavior change. These messages were designed to incorporate various behavior change techniques (BCTs) based on established theories, such as providing information about health consequences, using cues and prompts, and encouraging problem-solving. The selection of BCT was guided by a validated and reliable BCT taxonomy to ensure their effectiveness [26]. Multimedia Appendix 3 provides a comprehensive list of the current messages, along with the BCT they incorporate and whether they target motivation or volition. Moving forward, we intend to expand this message bank using the same principles and guidelines that were used to develop the existing messages.

Examples of MIRTH messages include the following:

1. “Physical activity is a great way to feel better overall, improve your cardiometabolic health, and lift your mood. Aim for at least 30 minutes of walking each day at a medium pace to see the benefits.”
2. “Going for a walk can improve your mood and clear your mind.”
3. “Can you find 30 minutes in your day to go for a walk? That is less time than it takes to watch one episode of a TV show.”
4. “Do not worry if you are not as physically active as others. Focus on you and your own goals. Everyone is different.”

We will track participants’ activity using the Fitbit Inspire 2 wrist-worn device, which connects to a smartphone through Bluetooth and collect the data using our custom-built LivePulse research app, available only in Australia on the App Store [27] (Apple) and Play Store [28] (Google).

#### Study Duration

The participant time line consists of 2 intervention periods lasting for 90 continuous days, spanning 4-6 months and 10-12 months (Figure 2). Throughout these intervention periods, participants will be required to wear Fitbit Inspire 2 wrist-worn devices continuously. Baseline data collection will take place for 1 month at month 3 before initiating the intervention for the 4-6–month study period. Another baseline data collection will occur at month 9 (natural baseline) for the 10-12–month study period.

**Figure 3:**
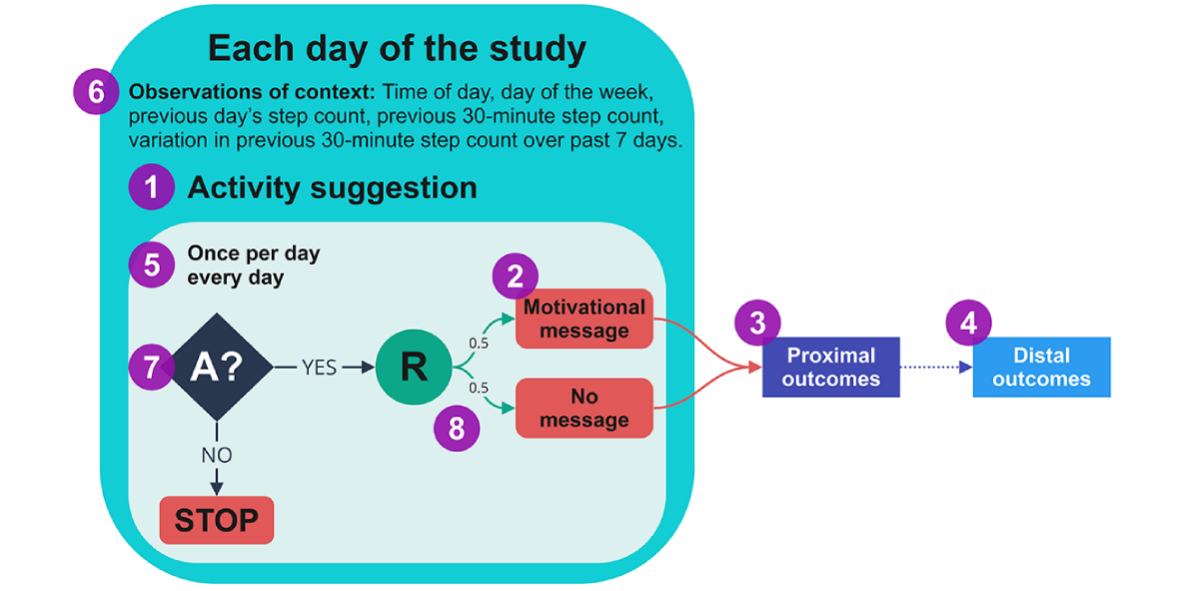
Each day of the Messages Improving Resting Heart Health trial. Please follow the numbers in the figure caption to guide you: (1) intervention component: activity suggestion sent as push notifications to participants’ smartphones as motivational messages. (2) Intervention options: motivational message (encouraging physical activity) or no message. (3) Proximal outcomes: physical activity duration, total step count, and heart rate, in the 180 minutes following a decision time point. (4) Distal outcomes: total step count and the change of resting heart rate during each 90-day study period. (5) Decision points: 6 AM, 11 AM, or 3 PM. (6) Observations of context: time of the day, day of the week, previous day’s step count, previous 30-minute step count, variation in previous 30-minute step count over past 7 days. (7) Availability: participants are deemed to be always available between 6 AM and 6 PM during the 90-day period. (8) Randomization probabilities: participants who are available at a decision point are randomized with a 0.5 probability to (a) receive or (b) not receive a motivational message.

Motivational messages will be randomly delivered to participants allocated to receive the intervention for that day at 3 different time points: 6 AM, 11 AM, or 3 PM (Figure 1). In the MIRTH study, specific exercises will not be prescribed through these messages. Participants have the freedom to choose the type, duration, and intensity of their physical activity. However, through the LIVEPLUS program, participants will receive a home-based program that aligns with the guidelines provided by the American College of Cardiology Foundation and the American Heart Association [29]. The following recommendations will be emphasized: (1) engaging in 30-60 minutes of moderate-intensity aerobic exercise, such as brisk walking, for at least five days, ideally 7 days per week; (2) enhancing daily physical activity through lifestyle activities (eg, taking walks during work, gardening, or household chores); and (3) incorporating resistance training into their routine at least two days per week. The delivery of messages will be monitored using app analytics to ensure successful delivery to participants (Figure 3).

### Outcome Measures

#### Primary Outcome

The number of steps walked within 180 minutes after either the message or no-message intervention is sent randomly at each decision-making time point (proximal).

#### Secondary Outcomes

The secondary outcomes are as follows: (1) the duration of time spent engaged in physical activity within 180 minutes after randomization at each decision-making time point (proximal short-term effect); (2) the change in heart rate within the next 180 minutes after receiving either the intervention or no intervention randomly (proximal short-term effect); (3) the average change values of resting heart rate within and across both phases 1 and 2 (distal long-term effect); and (4) the total number of steps walked within and across both phases 1 and 2 periods (distal long-term effect).

For the proximal analysis, which is based on an MRT design where each participant receives either control or intervention randomly at each selected decision time point over the study period, the proximal effect size of intervention (with-intervention vs without-intervention) is estimated. For the distal analysis, there is no control group; we only observe the total step count (total step counts accumulated over each phase) difference between phases 1 and 2 for each participant.

### Statistical Analysis

The effect sizes of the proximal outcome variables will be determined based on improvements in the number of steps (hypothesis 1), physical activity duration, and heart rate (hypothesis 2) observed within 180 minutes following the randomization of messages at each decision time point. To estimate these effect sizes, we will use the weighted and centered least-squares method as proposed by Boruvka et al [30]. This approach is specifically designed for analyzing longitudinal data within the context of an MRT design [20]. The weighted and centered least-squares method used in this study is similar to multilevel models, which account for the nested structure of the data, with daily time points nested within participants. It also shares similarities with the generalized estimating equation method. While there is a within-participant correlation across time in the outcome, the independent working correlation matrix is considered, as the message intervention is a time-dependent factor. This approach leverages sequential randomization to estimate the causal effects of the intervention. The estimated intervention effects are robust and not biased, even when covariates are included to reduce the variance of the estimates.

The regression model used in this study includes several covariates: days in study (ranging from 1 to 90 days), message type (intervention messages versus control), and an interaction term between days and message type. The message variable will be converted into a binary variable (0 for control and 1 for intervention), and it will be centered using the corresponding randomization probabilities (eg, A-0.5, where A represents the binary message variable, with A=1 for intervention and A=0 for control). Different trends over days, such as constant, linear, or quadratic, can be considered to assess the proximal effect of intervention messages in the regression models.

To estimate the proximal effect sizes of messages at different time points (hypothesis 3), we will replace (A-0.5) by (A-0.5)I_6AM_, (A-0.5)I_11AM_, and (A-0.5)I_3PM_ in the regression model. Here, I_6AM_=1 indicates that the participant is randomized to the 6 AM time point, and 0 indicates otherwise.

Our goal is to examine the effect of messages at these specific time points (6 AM, 11 AM, or 3 PM), considering that messages are only received at 1 of the 3 time points. Therefore, assuming a constant trend, we can model the daily steps using the following equation:

Y = β_0_ + β_1_(A-0.5)I_6AM_ + β_2_(A-0.5)I_11AM_ + β_3_(A-0.5)I_3PM_

Where Y=steps; β_0_ is the expected number of steps; and β_1_, β_2_, and β_3_ are the proximal effects of messages on steps at 6 AM, 11 AM, and 3 PM, respectively.

The effect size of the distal outcome variables will be assessed using a 2-tailed paired *t* test, assuming that there will be no significant difference in the long-term outcomes between phase 1 and phase 2 of the study (hypothesis 4).

### Sensitivity Analyses

Sensitivity analyses will be conducted for hypotheses 1-3, considering the following factors:

1. The intervention’s proximal effect size will be estimated by adjusting for additional baseline characteristics such as age, gender, and physical activity history. These characteristics can be included as covariates in the regression models.
2. The regression model estimating the proximal effect of the intervention will be based on participants who have at least 45 (50%) out of 90 days of data available.

In the case of positively skewed distributions of the primary outcome variables, the data will be transformed using logarithm or square root transformations. Before the transformations, a small decimal number (eg, 0.5) will be added to the zero values. This is done to address the issue of zero values in the data [20]. Missing data will be addressed using the full-information maximum likelihood approach [31].

### Power

Sample size calculations were conducted using Liao et al [32] as a reference, and an R-shiny app [33] was used for this purpose. Based on the calculations, a sample size of 58 participants was determined for randomization over a 90-day period (Multimedia Appendix 4). The calculations were performed with 80% power and a significance level of 5%. For the proximal outcomes, which involve 1 decision time point per day over the 90-day study duration and a randomization probability of 0.5, it is anticipated that participants will have an average availability rate of 0.8 at each decision time point, following a constant trend. The proximal effect size is expected to follow a quadratic trend, with an initial value of 0 and an average value of 0.1. The maximum value is anticipated to occur on the 45th day. For the distal outcome, such as the change in resting heart rate within either phase 1 or phase 2, the sample size of 58 participants can detect a standardized effect size of 0.374 (small to medium effect size) based on a paired *t* test.

### Data Exclusion

For activity suggestions, the interventional messages aim to promote physical activity, that is, walking. The primary outcome measure for each participant at each decision-making time point is the step count recorded within the subsequent 180 minutes after receiving a message. With 1 decision-making time point per day, this step count serves as the key data point for analysis. Detailed data management procedures have been outlined in the study’s protocol to ensure proper handling, processing, and analysis of the collected data [24].

### User Statistics

Fitbit data will be collected during participant visits, specifically scheduled to align with the 6th and 12th months of the LIVEPLUS. During these visits, the data from Fitbit devices will be downloaded and collated for further analysis (the time line described in Figure 2).

### Ethical Considerations

In Australia, all human research is reviewed by an independent human research ethics committee. MIRTH’s parent study LIVEPLUS received approval from the ethics review committee of the Royal Prince Alfred Hospital (RPAH) Zone of the Sydney Local Health District (protocol X20-0229 & 2020/ETH01273 on July 22, 2020). The original informed consent allows for secondary data analysis without additional consent. Before baseline testing, the research team will conduct a video call with potential participants to provide an overview of the study and address any questions or concerns. Any identifiable data obtained will remain confidential and encrypted on secure RPAH servers. Participants will be assigned a unique study ID number, and nonidentifiable data will be stored for at least 15 years. All deidentified data will be stored on university servers and only accessible by research staff through password-protected computers. The mobile app backs up data on both the smartphone and a web-based cloud server based in Sydney. While the app does not have a delete function, participants may request to have their data deleted from the cloud server. Access to mobile app data is restricted to the research team. There is no monetary compensation or amount for LIVEPLUS participants; however, participants who complete the 12-month period get to keep the Fitbit device used for physical activity monitoring.

## Results

The LIVEPLUS trial received funding in 2020 and initiated participant enrollment in February 2022. Recruitment for the MIRTH trial began in November 2022. As of September 2023, two participants have been enrolled in the MIRTH study and provided baseline data.

## Discussion

### Overview

The MIRTH trial aims to conduct a comprehensive analysis of the impact of a customized mHealth app on smart devices, delivering a lifestyle intervention to patients with coronary heart disease. This intervention includes time-based motivational messaging, which can be used to develop a JITAI. The goal of this approach is to provide prompts and cues at the optimal time to enhance motivation and promote increased physical activity behavior among participants. Through this study, we aim to gain insights into the effectiveness of this intervention in improving motivation and achieving the desired objectives of the study.

During the 90-day study duration, participants may receive randomized physical activity suggestions at up to 90 time points. Considering both phase 1 and phase 2 of the trial, there is a maximum potential for 180 time points in total (90 days for each phase). Since we anticipate an equal distribution of intervention and control messages among participants (with a randomization probability of 0.5), it is statistically unlikely for any participant to receive the intervention at all 180 time points. However, we can use the two 90-day study phases to examine improvements in distal outcomes. Our analysis will involve comparing resting heart rate and step counts from baseline (months 3 and 9) to the end of each phase. Furthermore, we will assess distal outcomes by comparing monthly changes within a study phase and between the 2 phases. Based on our hypothesis, we anticipate that participants who receive the intervention messages will demonstrate an increase in physical activity behavior aligned with the recommended guidelines. Additionally, we expect to observe improvements in resting heart rate and overall cardiometabolic health at the 12-month time point. This study will serve as a foundation for integrating an MRT design into routine clinical digital health prescriptions, especially for populations where lifestyle behavior change facilitated by digital health interventions can improve or potentially reverse disease states. The physical activity data collected during the baseline time points are more likely to reflect habitual physical activity behavior. In future studies, the incorporation of a reinforcement learning algorithm can consider additional factors such as user location, local weather conditions, and undisclosed calendar information to deliver a personalized and gamified JITAI.

### Strengths

This study introduces an innovative strategy to address physical activity behavior in patients diagnosed with coronary heart disease. It examines the timing of messages within each individual and their impact on outcomes. The study implements a layered digital intervention with minimal participant burden. It spans a longer duration of 180 days, divided into two 90-day periods. Furthermore, to our knowledge, this is the first MRT study to use incremental recruitment specifically for this population.

### Limitations

The findings of this study may have limited generalizability to the broader population with cardiovascular disease due to the specific characteristics of the study participants. It is important to note that this study is not designed as a double-blind study, as both participants and researchers are aware of the content and frequency of the messages being delivered. However, the lack of blinding does not pose a significant disadvantage since the outcomes are objectively assessed through Fitbit data, minimizing potential bias in outcome evaluation. It should be noted that this study does not incorporate reinforcement learning in the algorithm, which limits our ability to examine time-varying states for potential moderation effects, such as participant activity levels on the intervention day or the preceding day. Given that the intervention component is delivered through personal electronic devices such as smartphones and Fitbit, it is important to acknowledge the potential for technical issues that could impact the reliability of step count records. These challenges may include supporting participants in resolving technical issues with limited in-person assistance. It is worth noting that, in certain instances, Fitbit devices have been known to register hand movements as step counts, which could introduce some measurement inaccuracies [34]. To address potential data collection issues, the research team and technical support will closely monitor and cross-validate the data collection process, ensuring any troubleshooting is promptly addressed. Participants will be informed if any data collection issues occur. The incremental recruitment approach used in this study limits the ability to make adaptive changes based on preliminary results from phase 1 to inform and improve the intervention in phase 2. This may impact the optimization of the intervention based on real-time feedback. The 180-minute window for assessing the effect of messages may not capture the true change in motivation. There could be delayed effects observed weeks after receiving the messages, such as lag effects. Finally, the lack of message sequencing or tailored content may result in participants who are already making progress receiving messages that target earlier stages of motivation.

### Conclusions

The outcome of this study will provide preliminary insights into the value and efficacy of motivational messages in promoting physical activity behavior among patients with coronary heart disease in a community setting. The results will provide important data on the effectiveness of delivering physical activity motivational messages through a research study app. If proven effective, this approach could be implemented to enhance personalized care strategies, including the use of digital health interventions, for patients with coronary heart disease.

## Appendix

Multimedia Appendix 1 MIRTH randomization algorithm.

Multimedia Appendix 2 Screenshots of the mobile App.

Multimedia Appendix 3 Messages for MIRTH.

Multimedia Appendix 4 Table 1. Sample size calculations (Liao et al. 2016) from the R-shiny app.

## Abbreviations

BCT: behavior change technique
JITAI: just-in-time adaptive intervention
LIVEPLUS: Lifestyle Vulnerable Plaque Study
mHealth: mobile health
MIRTH: Messages Improving Resting Heart Health
MRT: microrandomized trial
RPAH: Royal Prince Alfred Hospital

## Data Availability

The data sets generated during and/or analyzed during this study will be made available from the corresponding author on reasonable request.
