# Supplementary figures and images for: Testing the Effects of App-Based Motivational Messages on Physical Activity and Resting Heart Rate Through Smartphone App Compliance in Patients With Vulnerable Coronary Artery Plaques: Protocol for a Microrandomized Trial
Source: JMIR Res Protoc. 2023 Oct 2;12:e46082. doi: 10.2196/46082 (PMC10580140; doi:10.2196/46082)

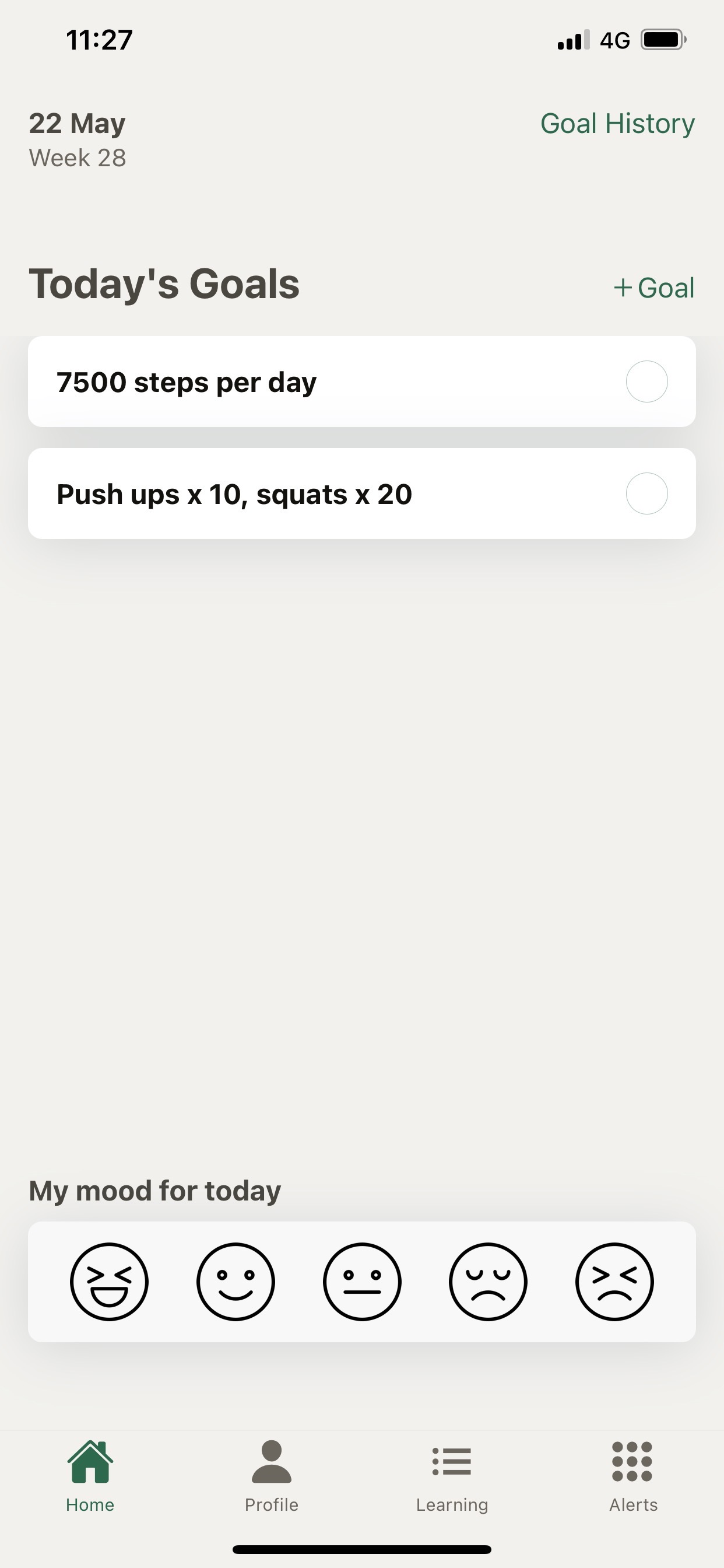

Supplement: Multimedia Appendix 2 [file resprot_v12i1e46082_app2.zip › Screenshot 1.JPG]

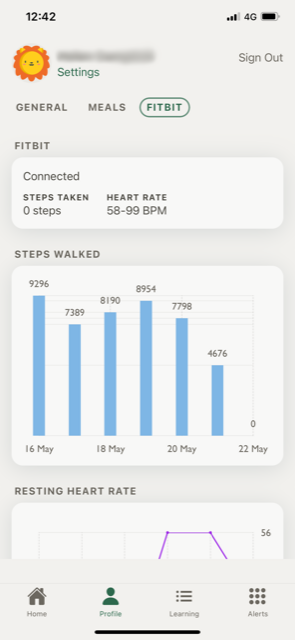

Supplement: Multimedia Appendix 2 [file resprot_v12i1e46082_app2.zip › Screenshot 2.PNG]

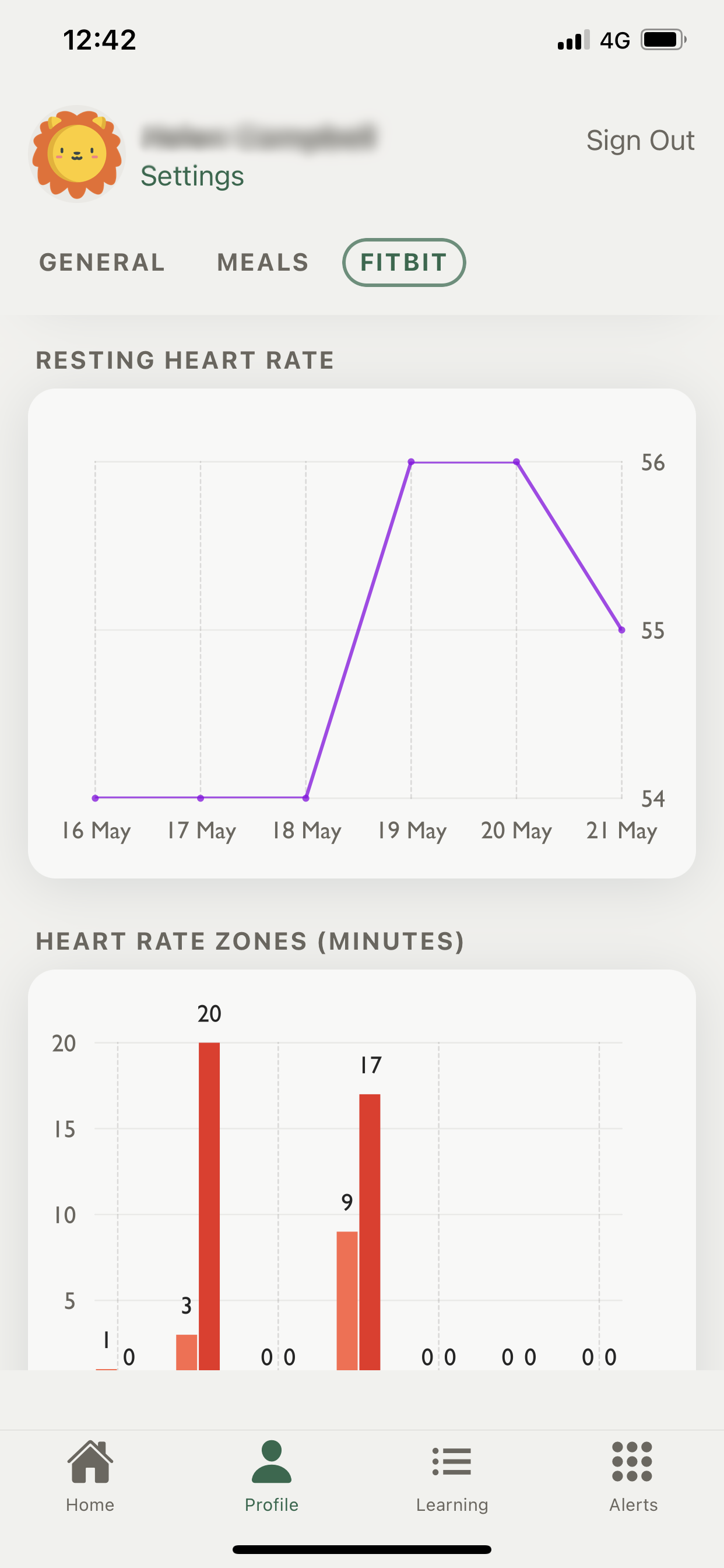

Supplement: Multimedia Appendix 2 [file resprot_v12i1e46082_app2.zip › Screenshot 3.PNG]

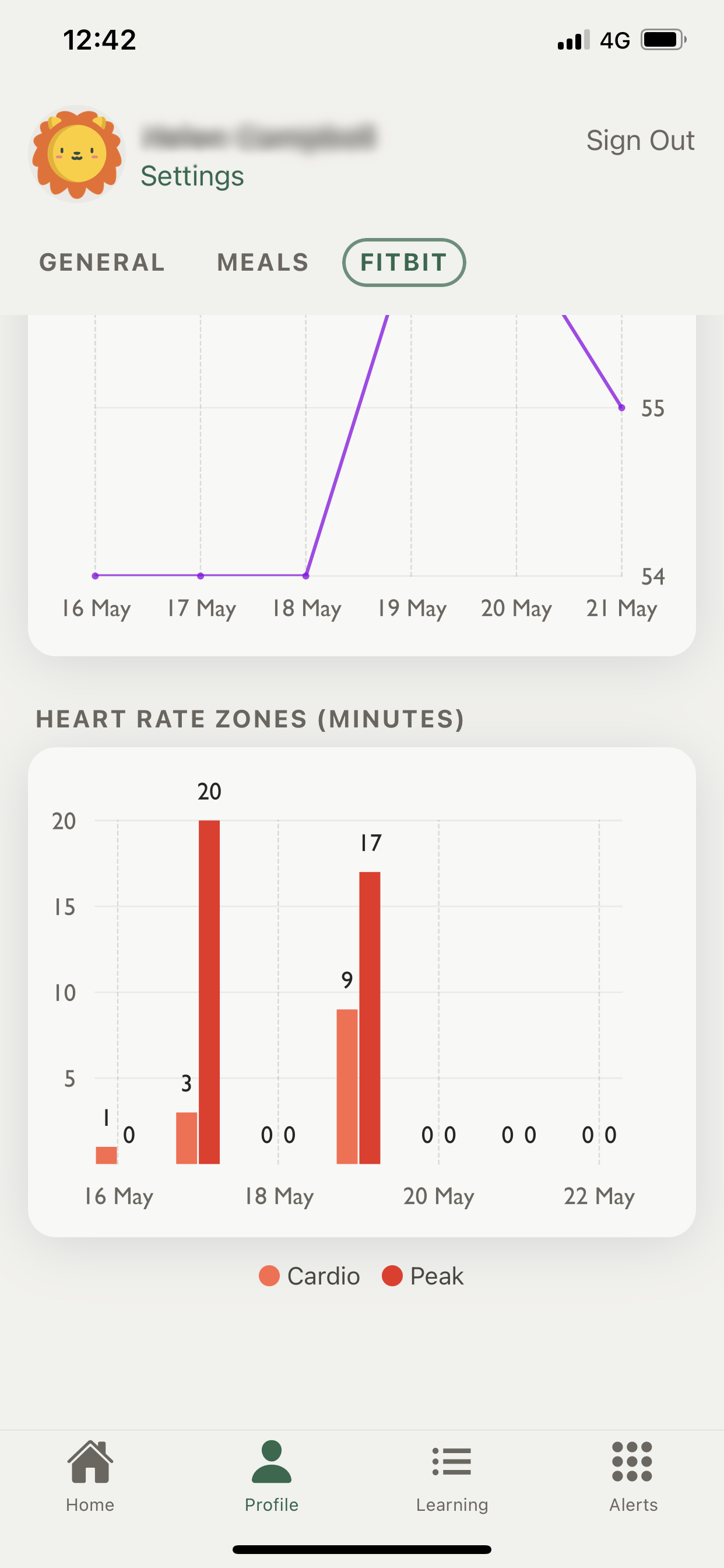

Supplement: Multimedia Appendix 2 [file resprot_v12i1e46082_app2.zip › Screenshot 4.PNG]

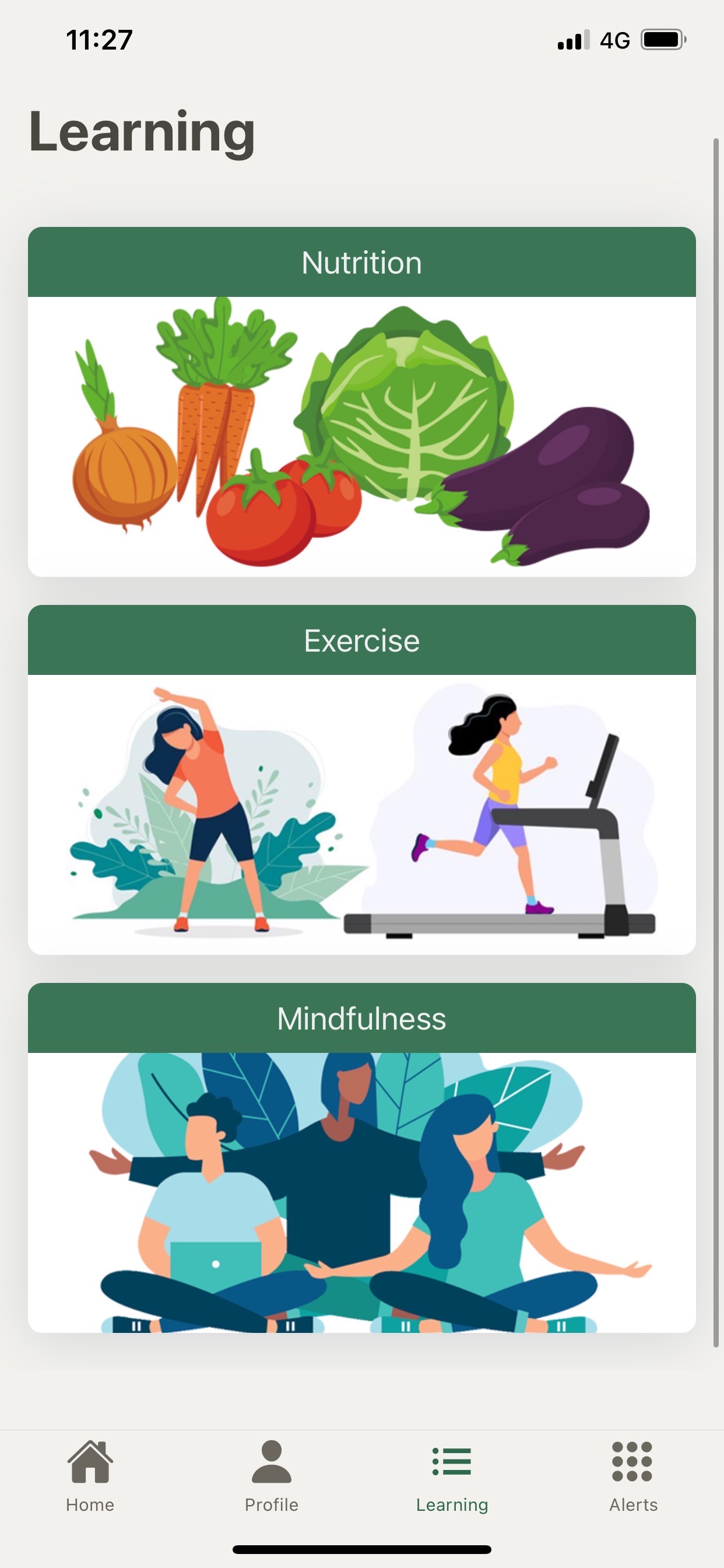

Supplement: Multimedia Appendix 2 [file resprot_v12i1e46082_app2.zip › Screenshot 5.JPG]

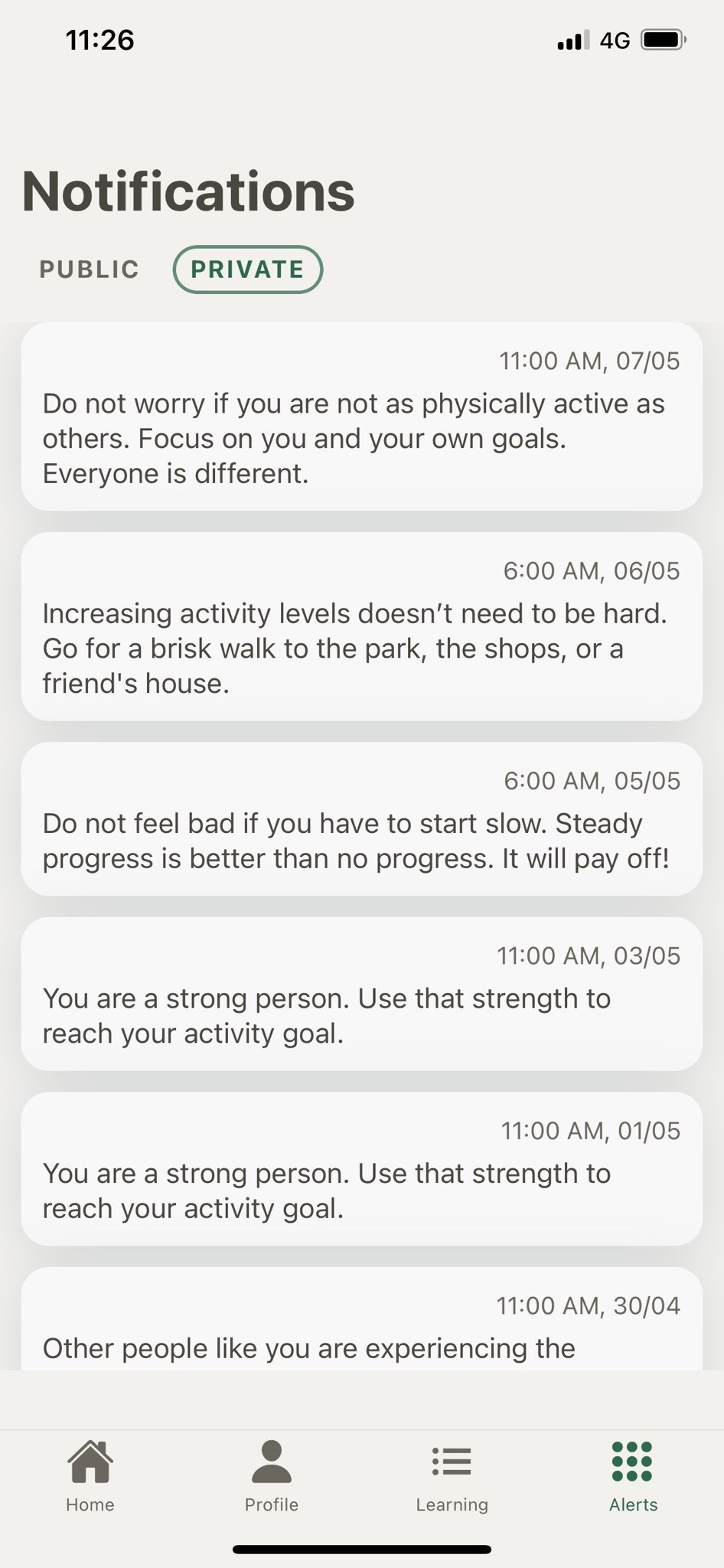

Supplement: Multimedia Appendix 2 [file resprot_v12i1e46082_app2.zip › Screenshot 6.JPG]
