# Supplementary material for: Testing the Effects of App-Based Motivational Messages on Physical Activity and Resting Heart Rate Through Smartphone App Compliance in Patients With Vulnerable Coronary Artery Plaques: Protocol for a Microrandomized Trial
Source: JMIR Res Protoc. 2023 Oct 2;12:e46082. doi: 10.2196/46082 (PMC10580140; doi:10.2196/46082)
Supplement: Multimedia Appendix 4 [file resprot_v12i1e46082_app4.docx]

## **Supplementary**

**Table 1.** Sample size calculations (Liao et al. 2016)^1^ from the R-shiny app.^2^

| Duration of study: | 90 days |
| --- | --- |
| Number of Decision Time Points per Day: | 1 |
| Constant Randomization Probability: | 0.5 |
| Expected Availability: | Constant |
| Average of Expected Availability: | 0.8 |
| Proximal Treatment Effect: | Quadratic |
| Average of Proximal Treatment Effect: | 0.1 |
| Day of Maximal Proximal Treatment Effect: | 45 |
| Initial value of Proximal Treatment Effect: | 0.02 |
| Desired Power: | 0.8 |
| Significance Level: | 0.05 |
| Required sample size: | 58 |

**References**

1. Liao P, Klasnja P, Tewari A, Murphy SA. Sample size calculations for micro-randomized trials in mHealth. *Statistics in Medicine*. 2016/05/30 2016;35(12):1944-1971. doi:10.1002/sim.6847

2. Seewald NJ, Sun J, Liao P. MRT-SS calculator: An R Shiny application for sample size calculation in micro-randomized trials. Accessed 08/06/2022, 2022. <https://pengliao.shinyapps.io/mrt-calculator/>
